# Effects of Bacterial Translocation and Autophagy on Acute Lung Injury Induced by Severe Acute Pancreatitis

**DOI:** 10.1155/2020/8953453

**Published:** 2020-02-13

**Authors:** Hanlin Wang, Chang Li, Yingjian Jiang, Hongbo Li, Dianliang Zhang

**Affiliations:** Center of Colon and Rectum, Qingdao Municipal Hospital, Qingdao University, No. 1 Jiaozhou Road, Qingdao, 266011 Shandong Province, China

## Abstract

**Aim:**

To reveal the role of bacterial translocation (BT) and autophagy in severe acute pancreatitis-induced acute lung injury (SAP-ALI).

**Methods:**

Rats were separated into a control (sham-operation) group (*n* = 10) and a SAP group (*n* = 10) and a SAP group (

**Results:**

Levels of TNF-*α*, IL-6, lipase, and amylase in the SAP group were significantly higher than those in the control group (*P* < 0.01). Histopathological score and W/D ratio of the lung in the SAP-BT(+) group were significantly higher than that in the SAP-BT(-) group (*P* < 0.01). Histopathological score and W/D ratio of the lung in the SAP-BT(+) group were significantly higher than that in the SAP-BT(-) group (*P* < 0.01). Histopathological score and W/D ratio of the lung in the SAP-BT(+) group were significantly higher than that in the SAP-BT(-) group (*P* < 0.01). Histopathological score and W/D ratio of the lung in the SAP-BT(+) group were significantly higher than that in the SAP-BT(-) group (*P* < 0.01). Histopathological score and W/D ratio of the lung in the SAP-BT(+) group were significantly higher than that in the SAP-BT(-) group (*P* < 0.01). Histopathological score and W/D ratio of the lung in the SAP-BT(+) group were significantly higher than that in the SAP-BT(-) group (

**Conclusions:**

BT can aggravate SAP-ALI with the increasing oxidative stress level, which may be related to the decrease of autophagy level.

## 1. Introduction

Severe acute pancreatitis (SAP) is a dangerous acute abdominal disease, which can cause systemic inflammatory response and rapidly accumulate multiple organs. Acute lung injury (ALI) is the most common and earliest organ with dysfunction in SAP. The mortality rate is over 30%, and the mortality rate of elderly patients is higher [1, 2]. It is one of the main causes of death in early SAP patients [3–5]. At present, the research direction of SAP-induced acute lung injury (SAP-ALI) is mainly focused on inflammatory factor-mediated lung injury [6, 7]. Its complex pathological mechanism is not fully understood [8, 9], which leads to high mortality and huge economic burden [1, 10].

Previous studies have found that impaired intestinal tight junction (TJ) barrier of SAP leads to increased intestinal permeability, which is one of the pathways for intestinal bacteria to enter the blood circulation and causes bacterial translocation (BT) [11]. It has been found that lipopolysaccharide (LPS) can activate pulmonary inflammatory cells and cause diffuse lung tissue damage [12, 13]. The damaged lung tissue will produce a large number of reactive oxygen species (ROS), which further aggravates oxidative stress and leads to a vicious cycle [14]. ROS has been shown to be a trigger for autophagy [15], and normal autophagy may be an effective mechanism to protect cell survival.

Autophagy is a catabolic process in which damaged organelles and intracellular garbage are degraded to participate in intracellular homeostasis [16]. It is found that autophagy may play an important role in cell protection under stress [17, 18]; even studies have found that autophagy can attenuate LPS-ALI [19]. But little is known about the change of autophagy level in SAP-ALI.

This study is aimed at revealing for the first time the effect of BT and autophagy in SAP-ALI, so as to provide a new theoretical basis for further understanding of SAP-ALI.

## 2. Materials and Methods

### 2.1. Animal Model Establishment and Group

A total of 40 Wistar rats (healthy, adult, males, and weighing 230-260 g) were adapted to our laboratory over 3 weeks and were then randomly assigned into either the SAP group (*n* = 20) or the control group (sham-operation group) (*n* = 10). All experimental protocols were based on the National Institutes of Health Laboratory Animal Guidelines and approved by the Institutional Animal Care and Use Committee of Qingdao University.

The SAP-ALI rat model was constructed according to a previously published protocol [20]. Briefly, rats received an intraperitoneal injection of 3% pentobarbital sodium (50 mg/kg) for anesthesia. At laparotomy, the biliary pancreatic duct was clipped near the hepatic portal, and then, duodenal intubation was performed. In the SAP group, 4% sodium taurocholate (1 ml/kg, Sigma) was slowly injected into the biliopancreatic duct for 1 min, and the needle was left in place for 5 min. In the control group, the same amount of sterile saline was injected into the biliopancreatic duct. Histopathology of the pancreas and lung confirmed the success of the SAP-ALI model.

18 hours after SAP-ALI model induction, rats were anesthetized again. Blood was taken from the inferior vena cava and then divided into two microtubules. One microtubule was centrifuged at 4°C and 3 000 revolutions per minute for 5 minutes. The supernatant was kept in an Eppendorf tube at -20°C for analysis of serum lipase and amylase. Other blood components were collected in EDTA microtubules and kept at -20°C for bacterial DNA analysis. Finally, the ileum close to the cecum, pancreas, and lung tissue was fixed with 4% paraformaldehyde.

### 2.2. Detection of Serum TNF-*α*, IL-6, Lipase, and Amylase

The concentration of serum TNF-*α* and IL-6 was determined by the rat TNF-*α* ELISA kit (Abcam) and the rat IL-6 ELISA kit (Abcam). In accordance with the instructions of the manufacturer, the detection of serum lipase and amylase content was carried out by the Olympus AU600 automatic biochemical analyzer (Tokyo, Japan).

### 2.3. Bacteria Detection by 16S rDNA Sequencing

Isolated DNA was considered as a template for extracting bacterial DNA from blood samples, amplifying the hypervariable regions of the 16S rRNA gene. R1521-1539 and F1169-1187-GC were used as the universal primers. Negative and positive controls were repeated two times for avoiding false-positive results. Polymerase chain reaction (PCR) was performed by geothermal cycling process after adding DNA (100 ng) to the reactant. The band sizes of PCR products (≈500 bp) and the specific amplification of prokaryotic 16S rRNA gene were consistent. PCR-amplified primers were also used to sequence about 600 bp length reading. Using advanced BLAST searches, the results of 16S rRNA sequence were matched with those from GenBank and ribosomal database project (RDP) at the National Center for Biotechnology Information (NCBI).

### 2.4. W/D Ratio of the Lung and Histopathological Score

Lung tissue wet/dry weight ratio (W/D ratio) was used to assess the extent of pulmonary edema. Fixing intestinal, pancreatic, and lung samples in polyformaldehyde solution, then they were embedded in paraffin section (5 *μ*m), hematoxylin and eosin dye, and morphological observation by an optical microscope. The injury degree of the pancreas, small intestine, and lung was scored on the basis of previous criteria [21–23].

## 3. Immunofluorescence Detection of LC3

Immunostaining was carried out according to a previously described method [24], embedding sections (5 *μ*m) in PFA, drying them at 37°C, and boiling them in 2 mM ethylenediaminetetraacetic acid solution. At room temperature, PBS that contained 1% bovine serum albumin and 5% (weight/volume) normal goat serum were used to incubate tissues for 30 minutes. In tissue immunostaining, sections were incubated overnight with the first antibodies at 4°C: rabbit anti-LC3 (Abcam). After rinsing with PBS solution, sections were added Alexa Fluor goat anti-rabbit IgG (wavelengths of 488, USA), incubating sections for 30 minutes and staining nuclei with DAPI for 5 minutes, acquiring pictures of lung tissue with an IX71 fluorescence inverted microscope (Olympus, Japan). ImageJ was used to analyze fluorescence intensity.

### 3.1. Western Blot Analysis

Proteins were extracted from the lung, determining their concentrations with a BCA Protein Assay Kit, separating them with a 10% sodium dodecyl sulfate-polyacrylamide gel electrophoresis, transferring them to a polyvinylidene fluoride membrane at room temperature for 1 hour, and incubating the membrane at 4°C overnight using first antibodies: rabbit anti-LC3 (Abcam), rabbit anti-Beclin1 (Abcam). The loading control was *β*-actin (Sigma). Incubating the membrane using second antibodies (GE Healthcare) at room temperature for 1 hour, densitometry determination of western blot bands was carried out through Image-Pro plus 6.0 software (Media Cybernetics, Rockville, MD, USA).

### 3.2. Malondialdehyde (MDA), Glutathione Peroxidase (GPx), and Superoxide Dismutase (SOD) Determination

Lung tissue is homogenized in a low temperature homogenizer. The homogenate is divided into two parts: one portion was measured for malondialdehyde (MDA) levels. The other portion was centrifuged, collecting the supernatant and assaying it for activity of GPx and SOD. And detection kits (Jiancheng Institute of Biotechnology, Nanjing, China) were used to measure them.

### 3.3. Statistical Analysis

SPSS version 24 (SPSS, Chicago, IL, USA) was used to analyze data. The data are normal distributed and apply the parametric analyses. From not less than three independent experiments, the data were used as the mean ± SD. One-way ANOVA and Dennett's *t* test were used to analyze differences among three groups (*P* < 0.05, statistically significant).

## 4. Result

### 4.1. Detection of BT and Intestinal Injury in SAP

**Figure 1:**
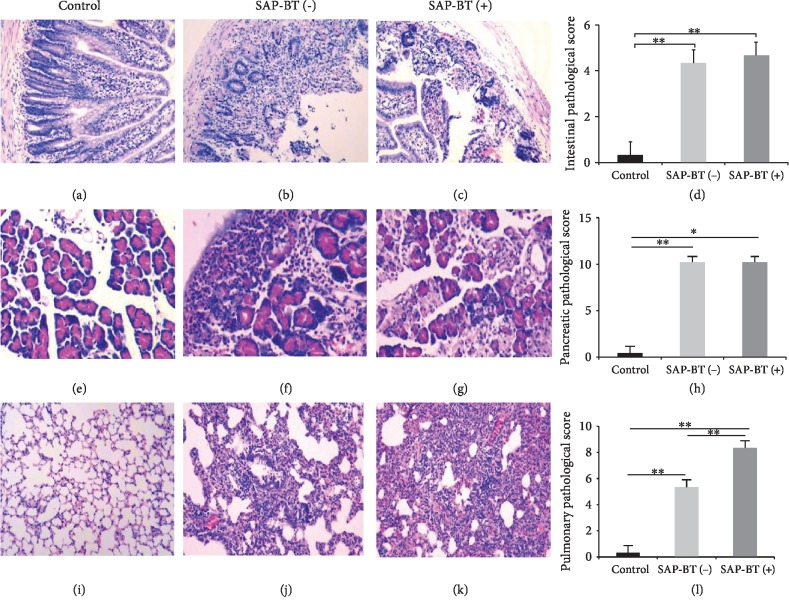
Representative HE sections of intestine, pancreas, and lung tissues. The control group of intestine (a), pancreas (e), and lung (i) tissues; the SAP-BT(-) group of intestine (b), pancreas (f), and lung (j) tissues; the SAP-BT(+) group of intestine (c), pancreas (g), and lung (k) tissues. Comparison of histological evaluation of the intestine (d), pancreas (h), and lung (l). Magnification, ×200. Results are expressed as the mean ± SD (*n* = 3, each). ^∗∗^*P* < 0.01.

After 18 hours of establishing the SAP rat model, 10 rats died. We used 16S rDNA sequencing technology to detect bacterial DNA in blood samples of 20 living SAP rats. Bacterial species from the rat blood are shown in Table 1. There were no BT in the control group, and the BT rate was 40% in the SAP group. According to the results of BT, rats were divided into a control group (*n* = 10), a SAP-BT(-) group (*n* = 12), and a SAP-BT(+) group (*n* = 8). As shown in Figures 1(a)–1(d), the degree of intestinal injury in the SAP group was significantly higher than that in the control group (*P* < 0.01). There was no significant difference between the SAP-BT(-) group and the SAP-BT(+) group (*P* > 0.05).

### 4.2. The SAP-ALI Model Was Successfully Constructed

**Figure 2:**
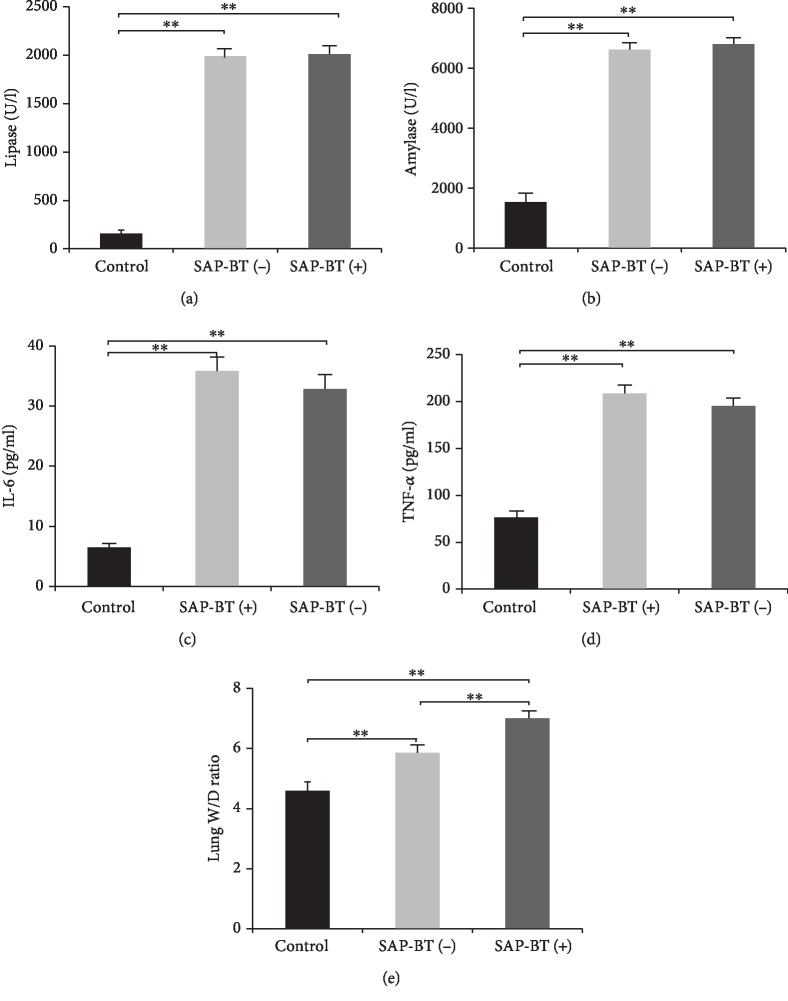
Levels of TNF-*α*, IL-6, lipase, and amylase and lung W/D ratio. Levels of lipase (a), amylase (b), IL-6 (c), and TNF-*α* (d) are significantly higher in the SAP group than those in the control group, and there are no significant difference between the SAP-BT(-) and the SAP-BT(+) groups. Lung W/D ratio (e) was the highest in the SAP-BT(+) group and the lowest in the control group. Results are presented as the mean ± SD (*n* = 3, each). ^∗∗^*P* < 0.01.

At the same time, we measured the level of TNF-*α*, IL-6, lipase, and amylase in rat blood. As shown in Figures 2(a)–2(d), levels of TNF-*α*, IL-6, lipase, and amylase in the SAP group were significantly higher than those in the control group (*P* < 0.01). The histopathological scores of the pancreas and lung are shown in Figures 1(e)–1(l): the degree of their injury in the SAP group was significantly higher than that in the control group (*P* < 0.01). And the result of lung W/D ratio was consistent with the histopathological score of the lung (*P* < 0.01) in Figure 2(e). These results indicate that the SAP-ALI model has been successfully constructed.

### 4.3. BT Can Aggravate SAP-ALI

**Figure 3:**
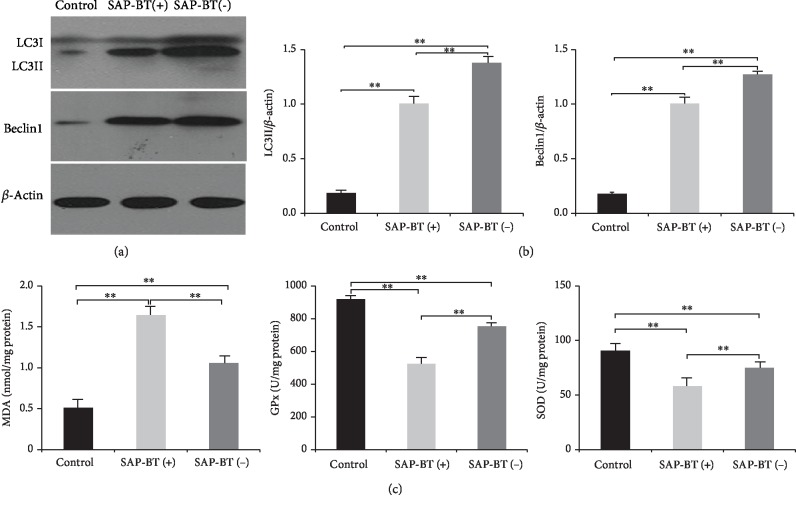
Representative western blot analysis of Beclin1 and LC3 of lung tissue and MDA content, GPX, and SOD activity. The expression of Beclin1 and LC3 is determined by optical densitometry in the control, SAP-BT(+), and SAP-BT(-) groups (a, b). (c) represents MDA content, GPX, and SOD activity in the lung tissue of the control group, the SAP-BT(+) group, and the SAP-BT(-) group. Results are expressed as the mean ± SD (*n* = 3, each). ^∗∗^*P* < 0.01.

Next, we analyzed the differences of data between the SAP-BT(-) group and the SAP-BT(+) group. As shown in Figures 1(e)–1(l), there was no significant difference in pancreatic tissue score between the two groups (*P* > 0.05), but the degree of lung injury in the SAP-BT(+) group was significantly higher than that in the SAP-BT(-) group (*P* < 0.01). And the result of the lung W/D ratio was consistent with the score of lung injury (*P* < 0.01) in Figure 2(e). In addition, there were no significant differences in serum IL-6 and TNF-*α* levels between the two groups in Figures 2(c) and 2(d). However, as shown in Figure 3(c), the activities of SOD and GPx in the SAP-BT(+) group were significantly lower than those in the SAP-BT(-) group (*P* < 0.01), while the content of MDA was significantly higher (*P* < 0.01). These results suggest that BT aggravates SAP-ALI with the increasing oxidative stress level.

### 4.4. Change of Autophagy Level in SAP-ALI

**Figure 4:**
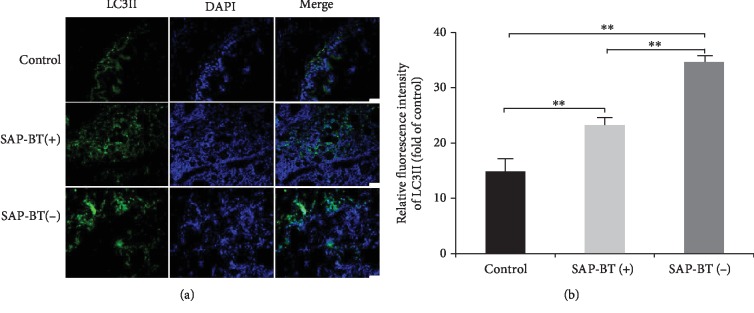
Immunofluorescence detection of LC3. Lung sections from the control, SAP-BT(+), and SAP-BT(-) groups (a) were stained with rabbit anti-LC3 (green), staining nuclei with DAPI (blue). The fluorescence intensity of LC3 was analyzed (b). Magnification ×200. Scale bar = 100 *μ*m. Results are presented as the mean ± SD (*n* = 3, each). ^∗∗^*P* < 0.01.

Autophagy has been found to enhance the removal of ROS and damaged mitochondria in cells, thus reducing oxidative stress [24]. So we further analyzed the differences in autophagy-related proteins between the SAP-BT(-) group and the SAP-BT(+) group. As shown in Figures 3(a) and 3(b), the expressions of LC3II and beclin1 were higher in the SAP-BT(-) group than those in the SAP-BT(+) group (*P* < 0.01). The results were consistent with those of LC3 immunofluorescence assay, as shown in Figures 4(a) and 4(b). And as mentioned above, the score of lung injury and result of lung W/D ratio were contrary to the expression of autophagy proteins. These results suggest that BT can aggravate SAP-ALI, which may be related to the decrease of autophagy level.

## 5. Discussion

The main cause of death in SAP patients is multiple organ dysfunctions (MODS), and ALI is the most common and earliest organ dysfunction in MODS. In this study, we used previously reported methods to establish the SAP-ALI model to observe lung injury in rats [20]. Animal and clinical studies of SAP have found that intestinal permeability is associated with the severity of the disease [25, 26] and may be accompanied by intestinal BT [27]. We verified the intestinal barrier damage in the SAP-ALI rat model from the histopathological level. And the rate of BT in our SAP-ALI rat model was 40%, which was similar to the previous report [28].

Oxidative stress reflects the excessive production of ROS and the decrease of scavenging ability of organisms to active intermediates, which is manifested by the decrease of activities of enzymes scavenging reactive oxygen species (such as SOD and GPX), the formation of malondialdehyde (MDA), and the damage of cell components [29]. Some studies have found that antioxidant therapy can alleviate acute lung injury [30]. Our results confirm that during SAP-induced acute lung injury, the oxidative stress index does change, that is, the more severe the lung injury, the lower the activity of SOD and GPX, and the higher the content of MDA.

Systemic inflammatory response can cause SAP-ALI [31], but it is not the only factor determining the severity of lung injury. Some studies have found that sepsis or bacterial endotoxin can cause acute lung injury [32–34]. Our results also confirm the above view in SAP-ALI. Compared with the control group, SAP can cause acute lung injury. But there were no differences in the inflammatory factors represented by IL-6 and TNF-*α* in the SAP-BT(+) group and the SAP-BT(-) group. The degree of lung injury in the BT(+) group is more serious than that in the BT(-) group, and the level of oxidative stress is higher. This suggests that BT may aggravate SAP-ALI with the increasing oxidative stress level.

Autophagy can clearly damage mitochondria [35], and damaged mitochondria are the main source of intracellular ROS [29]. Some studies have found that autophagy reduces oxidative stress by promoting the removal of reactive oxygen species in cells [24]. Therefore, we observed the change of autophagy level through bacterial translocation. Compared with the BT(-) group, the autophagy level in the BT(+) group is lower, which indicates that autophagy may be affected by BT, which in turn affects the level of oxidative stress. But the clear relationship between BT and autophagy needs further study.

Our results showed that there was no statistical difference in the pathological scores of the pancreas and intestine between in the SAP-BT(-) group and in the SAP-BT(+) group, and there was no statistical difference in the levels of serum amylase, lipase, IL-6, and TNF-*α*, which indicated that the severity of SAP disease was similar between in the SAP-BT(-) group and in the SAP-BT(+) group. However, there were statistically significant differences in the lung pathological score and lung wet/dry ratio between in the SAP-BT(-) group and in the SAP-BT(+) group, so we think that bacterial translocation caused the difference in lung injury between the two groups. The results of this experiment show that bacterial translocation is expected to become an indicator of SAP severity, which needs further research to verify, such as the application of the SAP-ALI model of sterile animals.

Our study focused for the first time on the role of BT in exacerbating SAP-ALI with the increasing oxidative stress level, which may be related to the decrease of autophagy level. Understanding the effects of BT and autophagy on SAP-ALI may provide a reference for new therapeutic strategies.

## Figures and Tables

**Table 1:** Bacteria species identified in SAP-BT(+) rat blood samples.

Rats no.	Bacterial species	Rats no.	Bacterial species	Rats no.	Bacterial species
1	Enterococcus aerogenes	13	Streptococcus pneumonia	25	Citrobacter freundii
6	Escherichia coli	17	Enterococcus faecium	29	Escherichia coli
9	Enterococcus aerogenes	23	Prevotella copri		

## Data Availability

The data used to support the findings of this study are available from the corresponding author upon request.
